# Foot‐Pedal Monitoring Camera Improves Pedal Operation During Simulated Therapeutic Endoscopy

**DOI:** 10.1002/deo2.70452

**Published:** 2026-10-14

**Authors:** Riku Nakajima, Wataru Sano, Sachiko Shimizu, Daizen Hirata, Mineo Iwatate, Mikio Fujita, Yasushi Sano

**Affiliations:** ^1^ Gastrointestinal Center and Institute of Minimally‐Invasive Endoscopic Care (iMEC) Sano Hospital Hyogo Japan

**Keywords:** endoscopy, endoscopic submucosal dissection, equipment design, ergonomics, simulation training

## Abstract

**Objectives:**

Therapeutic endoscopic procedures often require the coordinated use of multiple foot pedals, making pedal operation challenging. We developed a foot‐pedal monitoring camera system that displays the pedal area on the endoscopic monitor, allowing operators to confirm pedal position without looking away from the procedural field. This study evaluated the effect of the system on foot‐pedal operation during simulated therapeutic endoscopy.

**Methods:**

This single‐center simulation study used a custom‐built simulator that displayed one of four commands on a monitor: cutting, coagulation, water jet, or injection. Participants responded by pressing the corresponding foot pedal. Each participant completed three sets of 20 commands under two conditions: with and without camera assistance. The primary outcome was participant‐level mean reaction time. Secondary outcomes included participant‐level median reaction time, incorrect pedal presses, and gaze shifts toward the foot pedals.

**Results:**

Eighteen endoscopists from 13 institutions completed all simulation tasks under both study conditions. Camera assistance significantly reduced the mean reaction time compared with the conventional condition (1190.4 ms vs. 1280.8 ms; *p* = 0.0024). Participant‐level median reaction times were also significantly shorter with camera assistance. In addition, camera assistance reduced incorrect pedal presses and gaze shifts.

**Conclusions:**

The foot‐pedal monitoring camera improved the speed of pedal operation and reduced incorrect pedal presses and gaze shifts during simulated therapeutic endoscopy. Further clinical investigation is needed to determine whether these findings translate into improved pedal operation during actual therapeutic endoscopy.

**Trial Registration**: University Hospital Medical Information Network: UMIN000060763

## 1 Introduction

Endoscopic submucosal dissection (ESD) is an effective technique for achieving en bloc resection; however, it requires considerable technical expertise. Operators must maintain a stable endoscopic view while precisely manipulating therapeutic devices [1, 2]. During critical procedural steps, including mucosal incision, submucosal dissection, and hemostasis, operators often have only a limited opportunity to treat the target lesion while maintaining both a stable endoscope position and adequate visualization. Consequently, they must promptly select the appropriate device and initiate treatment without delay. Even brief delays may result in deterioration of the visual field, requiring repositioning of the endoscope or reconstruction of the visual field, thereby prolonging the procedure, particularly for less experienced endoscopists.

Recent advances in therapeutic endoscopy have increased the number of foot‐operated functions, further complicating foot‐pedal operation during ESD. In underwater ESD using the water‐pressure method, maintaining adequate procedural visualization requires timely activation of the water‐jet function and rapid hemostasis, making accurate foot‐pedal operation particularly important [3, 4]. Operators must simultaneously control multiple foot pedals while maintaining a stable endoscopic view, and looking away from the monitor to confirm pedal position may disrupt procedural flow and increase physical strain. Because therapeutic endoscopy requires the simultaneous management of the endoscopic view, scope control, device manipulation, and peripheral equipment, the work environment and equipment layout may influence operator workload and increase the risk of endoscopy‐related musculoskeletal disorders [5, 6, 7].

Displaying the foot‐pedal area on the endoscopic monitor may enable operators to identify pedal positions without diverting their attention from the procedural field. In surgical settings, foot‐pedal monitoring has been investigated as a strategy to assist with the identification and operation of multiple pedals [8]. However, evidence supporting its use in therapeutic endoscopy, particularly during ESD, remains limited. Based on challenges encountered during routine ESD practice, we developed a foot‐pedal monitoring system that allows operators to confirm pedal positions while maintaining their gaze on the procedural monitor. Our initial clinical experience with the system led us to hypothesize that it might reduce gaze shifts and incorrect pedal presses. However, these potential benefits would have limited practical value if they were achieved through slower, more cautious pedal operation. We therefore selected mean reaction time as the primary outcome and also evaluated incorrect pedal presses and gaze shifts. We compared pedal‐operation performance with and without camera assistance.

## 2 Methods

### 2.1 Study Design and Setting

This single‐center simulation study evaluated the effect of a foot‐pedal monitoring camera positioned above the foot pedals on foot‐pedal operation. The study was conducted at our institution. Participants were endoscopists recruited from 13 institutions during a training seminar held at our hospital.

Each participant completed the task under both conditions—with and without camera assistance— in randomized order.

Before participation, all participants provided written informed consent. The study protocol was approved by our institutional ethics committee (Approval No. 202602‐02). The study was registered with the University Hospital Medical Information Network Clinical Trials Registry (UMIN000060763).

### 2.2 Participants

Participants were endoscopists with experience performing endoscopic procedures using foot pedals. The exclusion criteria included no prior experience using foot pedals for endoscopic treatment, missing data, incomplete video recordings, or technical issues that prevented accurate data collection; foot or lower‐extremity pain or motor impairment that interfered with foot‐pedal operation; withdrawal of consent; and any condition that the principal investigator considered unsuitable for participation.

### 2.3 Simulation Setup

All participants performed the tasks while standing. The monitor position, participant‐to‐monitor distance, and room lighting were maintained as consistently as possible. Each foot pedal measured 100 × 60 mm and was secured to the floor with a consistent distance maintained between adjacent pedals (Figure 1). The task was performed using a custom application developed for a personal computer and displayed on the endoscopic monitor of the EVIS X1 system (Olympus, Tokyo, Japan) to simulate the clinical display environment. Using this application, participants performed the foot‐pedal task under both conditions: with and without camera assistance.

**FIGURE 1:**
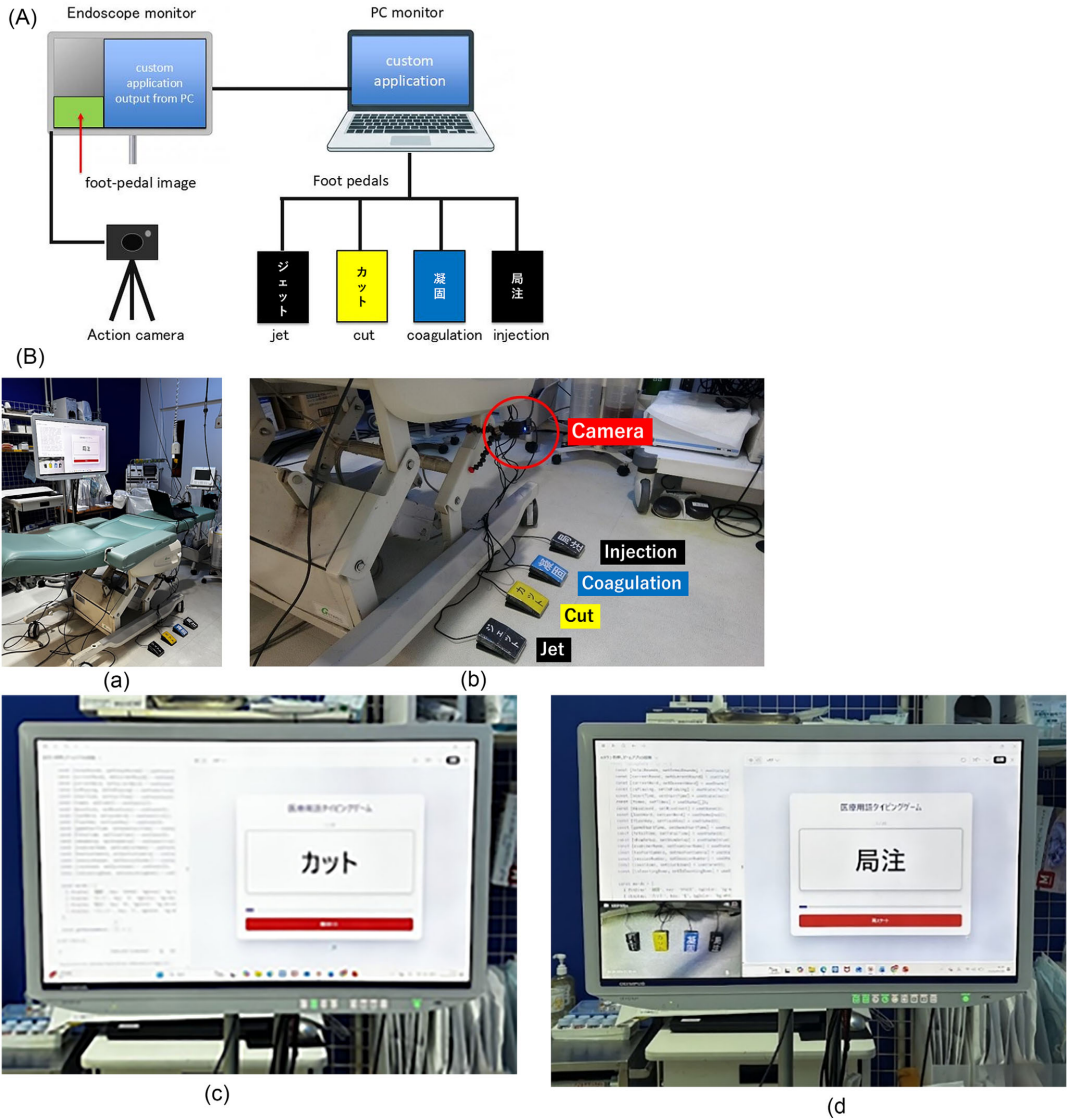
Experimental setup. (A) Schematic illustration of the experimental setup. A custom application running on a personal computer was displayed on the endoscopic monitor through a wired connection. An action camera was also connected to the endoscopic monitor, and the foot‐pedal image was displayed in the lower‐left corner of the screen in a picture‐in‐picture format. Four foot pedals were connected to the personal computer via wired connections and were labeled jet, cut, coagulation, and injection. (B) Actual experimental setup mimicking a clinical environment. (a) overview of the experimental setup, (b) four foot pedals with an overhead camera (red circle), (c) endoscopic monitor view without camera assistance, and (d) endoscopic monitor view with camera assistance. English labels corresponding to the Japanese text on the foot pedals (jet, cut, coagulation, and injection) are overlaid for clarity.

### 2.4 Study Procedure

In each condition, one of four command words (“cut,” “coagulation,” “jet,” or “injection”) was randomly displayed on the monitor. Reaction time was automatically measured from the appearance of the command word until the application registered activation of the correct corresponding pedal. If an incorrect pedal was pressed, the input was recorded as an incorrect pedal press, and measurement continued until the correct pedal was activated (Video S1). The foot used for pedal operation was not predefined or standardized. Participants were permitted to use the right foot, left foot, or both feet according to their usual practice during ESD. This policy applied to both study conditions and all trials. After a practice session, participants completed three sets of 20 trials under each condition. With camera assistance, an image of the pedal area was displayed in the lower‐left corner of the monitor in a picture‐in‐picture format, as in clinical practice; without camera assistance, this image was not displayed. In both conditions, participants were permitted, but not required, to look down at their feet.

The foot pedals were physically rearranged in a randomized order before each set. This procedure was applied in both study conditions to minimize confounding by learning and motor memory and to maintain conditions in which accurate pedal operation required position confirmation. Because incorrect pedal activation during actual ESD may lead to serious adverse events, participants were instructed to prioritize avoiding incorrect pedal presses and operating the pedals accurately. They were then instructed to respond as quickly as possible while maintaining that priority.

### 2.5 Outcome Measures

The primary outcome was the participant‐level mean reaction time. Secondary outcomes included the participant‐level median reaction time, incorrect pedal presses, and gaze shifts. For each participant, mean and median reaction times were calculated across the 60 trials separately for each condition. Participant‐level mean and median reaction times were summarized across participants as mean ± SD. An incorrect pedal press was defined as activation of a pedal that did not correspond to the command displayed on the monitor. Gaze shifts were defined as distinct movements of the head or face toward the foot pedals to verify pedal position. Reaction time and incorrect pedal presses were automatically recorded by the custom application. Gaze shifts were evaluated from lateral video recordings. Continuous orientation toward the foot pedals was counted as a single gaze shift, whereas repeated movements following a return to the forward‐facing position were counted as separate events.

### 2.6 Participant Enrollment

The required sample size was estimated based on findings from a pilot study involving seven expert endoscopists. In the pilot study, use of the foot‐pedal monitoring camera reduced the mean reaction time by 144.29 ms (*p* = 0.0096; Cohen's *dz* = 1.415). Because the pilot sample was small, the observed effect size was considered uncertain and potentially overestimated.

During study planning, in consultation with a statistician, we examined the required sample size under four scenarios. These scenarios combined a 20% (*dz* = 1.13) or 30% (*dz* = 0.99) attenuation of the observed effect size with a statistical power of 80% or 90%. These attenuation rates were specified as practical design assumptions to avoid basing the sample‐size calculation directly on the potentially overestimated effect size from the small pilot study. A 30% attenuation yielded *dz* = 0.99, approximately 1.0, indicating that the mean within‐participant difference was approximately equal to the standard deviation of the within‐participant differences. We considered this a reasonable and more conservative effect size than the very large pilot estimate. We selected the most conservative scenario, which assumed *dz* = 0.99 and 90% power.

With a two‐sided significance level of 0.05, the selected scenario indicated that at least 14 participants were required. To account for potential missing data and participant exclusions, the target enrollment was set at 18 participants. To enhance the generalizability of the findings, participants were recruited from multiple institutions.

### 2.7 Statistical Analysis

Analyses were based on within‐participant differences between the camera and non‐camera conditions. Participant‐level mean and median reaction times were compared between conditions using paired t tests. Incorrect pedal presses and gaze shifts were analyzed using the Wilcoxon signed‐rank test. For each participant, the mean reaction time in each condition was calculated by averaging the mean reaction times across the three sets. The reduction in mean reaction time was calculated as the mean reaction time under the non‐camera condition minus that under the camera‐assisted condition. All statistical tests were two‐sided, and a *p*‐value <0.05 was considered statistically significant. Statistical analyses were performed using EZR version 1.70.

## 3 Results

### 3.1 Study Population and Baseline Characteristics

A total of 18 endoscopists from 13 institutions were enrolled in this study. All participants completed both study conditions and were included in the final analysis. No participants were excluded, and no missing data were observed (Figure 2). Participant characteristics are summarized in Table 1. Among the participants, 13 (72.2%) were male, and the median endoscopic experience was 4 years [range, 2–24 years].

**FIGURE 2:**
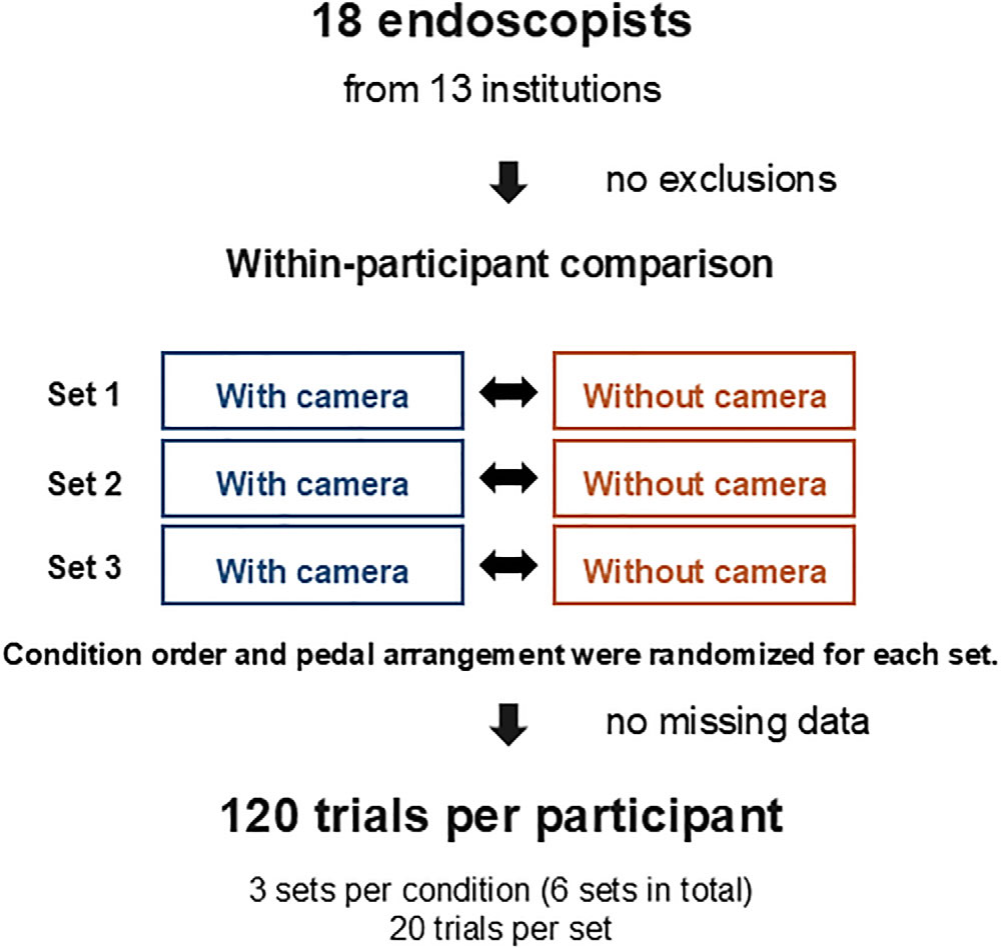
Study flow. Flow diagram illustrating participant enrollment, completion of both study conditions, and inclusion in the final analysis. No participants were excluded, and no missing data were observed.

**TABLE 1:** Baseline characteristics of the study participants.

Characteristic	Value
Participants, *n*	18
Institutions, *n*	13
Endoscopic experience, median [range], years	4 [2–24]
Endoscopic experience category, *n* (%)	
1–5 years	12 (66.7)
6–10 years	3 (16.7)
11–15 years	1 (5.6)
≥16 years	2 (11.1)
Sex, *n* (%)	
Male	13 (72.2)
Female	5 (27.8)

### 3.2 Primary Outcome

The mean reaction time was significantly shorter under the camera‐assisted condition than under the non‐camera condition (1190.4 ms vs. 1280.8 ms; *p* = 0.0024; Cohen's *dz* = 0.8394). The mean difference, calculated as the non‐camera condition minus the camera‐assisted condition, was 90.4 ms (95% confidence interval, 36.8–144.0 ms) (Table 2).

**TABLE 2:** Primary and secondary outcomes with and without camera assistance.

Outcome	Without camera assistance (*n *= 18)	With camera assistance (*n* = 18)	Difference (without − with)	*p*‐Value
Mean reaction time, ms	1280.8 ± 244.0	1190.4 ± 209.3	90.4 (95% CI, 36.8–144.0)	0.0024
Mean of participant‐level median reaction times, ms	1207.1 ± 234.3	1141.0 ± 208.0	66.1 (95% CI, 27.5–104.8)	0.0022
Incorrect pedal presses	1 [0–2]	0 [0–1]	NA	0.0378
Gaze shift count	59.5 [45.5–60.0]	0 [0–0.75]	NA	<0.001

Participant‐level mean reaction times and participant‐level median reaction times are presented as mean ± SD. Incorrect pedal presses and gaze‐shift counts are presented as median [interquartile range]. Participant‐level mean and median reaction times were analyzed using paired t tests, and their mean paired differences (without camera assistance minus with camera assistance) are presented with 95% confidence intervals. Incorrect pedal presses and gaze‐shift counts were analyzed using the Wilcoxon signed‐rank test.

Abbreviations: CI, confidence interval; IQR, interquartile range; NA, not applicable; SD, standard deviation.

### 3.3 Secondary Outcomes

The mean of the participant‐level median reaction times was significantly shorter under the camera‐assisted condition than under the non‐camera condition (1141.0 ms vs. 1207.1 ms; *p* = 0.0022) (Table 2). Compared with the non‐camera condition, the camera‐assisted condition resulted in significantly fewer incorrect pedal presses (0 vs. 1; *p* = 0.0378) and significantly fewer gaze shifts (0 vs. 59.5; *p* < 0.001).

## 4 Discussion

The primary finding of this study was that use of the foot‐pedal monitoring camera significantly reduced reaction time during simulated therapeutic endoscopy. This improvement was accompanied by fewer incorrect pedal presses and fewer gaze shifts toward the pedal area. Together, these findings indicate faster pedal operation with fewer incorrect inputs and fewer gaze shifts under the simulated experimental conditions.

The reduction in reaction time may be explained by the ability of operators to confirm pedal positions on the procedural monitor without looking away from the procedural field. Even experienced endoscopists who are familiar with a fixed pedal layout may need to visually reconfirm pedal positions during critical steps or when switching pedals. Without looking down, they may also locate a pedal by touch and confirm its shape before activation. Pedals may also shift from their initial positions during a procedure and require reconfirmation. The present study was designed to evaluate camera assistance in these situations. Consistent with this interpretation, camera assistance reduced gaze shifts and incorrect pedal presses. Because therapeutic endoscopy requires simultaneous monitoring of the procedural field, endoscope control, device manipulation, and foot‐pedal operation, minimizing unnecessary visual distraction may support smoother pedal operation.

The mean reaction time was reduced by 90.4 ms. The potential value of the system should not be interpreted on the basis of the 90.4‐ms reduction alone. Rather, it should be interpreted in light of the combined findings that camera assistance reduced gaze shifts and incorrect pedal presses without delaying pedal operation and, in fact, modestly shortened reaction time. These findings may be particularly relevant during technically demanding procedures such as ESD and underwater ESD, which frequently require rapid switching between cutting, coagulation, injection, and water‐jet functions. Prompt and accurate pedal activation may help maintain procedural continuity when stable visualization is critical. However, because this study was conducted in a simulated setting, further clinical studies are needed to determine whether these findings translate into improved pedal operation during actual therapeutic endoscopy.

This study has several limitations. First, this was a single‐center simulation study with a relatively small sample, and most participants were early in their endoscopic careers. Although the exploratory analysis showed no clear association between endoscopic experience and the reduction in mean reaction time (Spearman's *ρ* = −0.069, *p* = 0.785; *n* = 18), the small sample size and uneven distribution of endoscopic experience limited our ability to detect experience‐related differences. Therefore, the possibility that the effect of camera assistance may vary according to endoscopic experience cannot be excluded. Second, the experimental task presented the required function as a command word to standardize and isolate the subsequent pedal‐identification and activation process, which was intended to simulate the pedal‐operation phase after the required function had been determined during actual ESD. However, actual ESD requires pedal operation while simultaneously manipulating the endoscope and treatment devices and involves other clinical factors, such as bleeding, fibrosis, respiratory motion, and coordination with assistants. Therefore, procedural efficiency and safety in clinical practice were beyond the scope of this study. Third, foot‐pedal configurations were randomized before each set, whereas pedal layouts are generally fixed in routine clinical practice. The same randomization procedure was applied in both study conditions to minimize confounding by learned pedal configurations and motor memory. Although this design did not fully reproduce routine fixed layouts, the present study was intended to evaluate camera assistance in situations requiring pedal‐position confirmation. Fourth, the number of gaze shifts served as a surrogate measure of foot‐checking behavior and did not directly assess visual attention or cognitive workload. Fifth, the present simulation did not assess all practical limitations that may arise during clinical use. The foot‐pedal image displayed in the lower‐left corner does not obscure the main live endoscopic field but may partially overlap with stored still images displayed on the left side of the monitor. The effect of the simultaneous display on operator attention was not assessed in this study.

Despite these limitations, this study has several strengths. We developed a simulation environment designed to replicate the practical challenges of foot‐pedal operation during therapeutic endoscopy. This experimental setup enabled quantitative evaluation of reaction time, incorrect pedal presses, and gaze shifts. Furthermore, because each participant completed both study conditions, interindividual variability was minimized through a within‐participant study design.

In conclusion, use of the foot‐pedal monitoring camera improved the speed of pedal operation and reduced incorrect pedal presses and gaze shifts during simulated therapeutic endoscopy. Further clinical investigation is needed to determine whether these findings translate into improved pedal operation during actual therapeutic endoscopy.

### 4.1 Presentation

A preliminary version of this study was presented at the President's Special Session (JGES YOKOHAMA Cup) during the 111th Congress of the Japan Gastroenterological Endoscopy Society, held in Yokohama, Japan, in May 2026, under the title *“Utilization of a Foot‐Pedal Monitoring Camera in ESD.”* The presentation was awarded the Grand Prize.

## Author Contributions

**Riku Nakajima**: conceptualization; methodology; software; formal analysis; data curation; Writing – original draft; project administration. **Wataru Sano**: validation; supervision; writing – review & editing. **Yasushi Sano**: resources; supervision; writing – review & editing. **Sachiko Shimizu**, **Daizen Hirata**, **Mineo Iwatate**, and **Mikio Fujita**: investigation; writing – review & editing. All authors read and approved the final version of the manuscript.

## Funding

The authors have nothing to report.

## Ethics Statement

Approval of the research protocol by an Institutional Review Board: The study protocol was approved by the Institutional Review Board of Sano Hospital (Approval No. 202602‐02).

## Consent

Written informed consent was obtained from all participants.

## Conflicts of Interest

The authors declare no conflicts of interest.

## Supporting information




**Supporting File 1**: deo270452‐sup‐0001‐video.mp4.

**Supporting File 2**: deo270453‐sup‐0002‐SuppMat.docx.

## Data Availability

The data that support the findings of this study are available from the corresponding author upon reasonable request.
