# Supplementary material for: Foot‐Pedal Monitoring Camera Improves Pedal Operation During Simulated Therapeutic Endoscopy
Source: DEN Open. 2026 Oct 14;7(1):e70452. doi: 10.1002/deo2.70452 (PMC13669537; doi:10.1002/deo2.70452)
Supplement: Supplementary file 1 [file DEO2-7-e70452-s001.docx]

**Supporting Information Legends**

**Figure 1.**

A)

B)

(a)

(b)

(c)

(d)

**Figure 2.**

**Supplement video**

Video. Development of task application, construction of simulation environment, and representative task performance. The video demonstrates the custom application developed for this study, the construction of the simulation environment, and representative task performance with and without camera assistance.
